# A Case of a Metastatic Disease to the Pancreas from a Small-Cell Lung Carcinoma Documented by a CT-Scan-Guided Trucut Biopsy: The Diagnostic Role of Cytomorphology and Immunohistochemistry

**DOI:** 10.1155/2012/520430

**Published:** 2012-10-16

**Authors:** N. Bouyahia, K. Daoudi, K. Moumna, F. Z. Hijri, H. Benhammane, S. A. Brahmi, S. Arifi, N. Mellas, A. Amarti, O. El Mesbahi

**Affiliations:** ^1^Department of Medical Oncology, Hassan II University Hospital, Fez, Morocco; ^2^Department of Pathology, Hassan II University Hospital, Fez, Morocco

## Abstract

Unlike primary pancreatic carcinoma, metastatic lesions of the pancreas are uncommon and account for approximately 2% of pancreatic malignancies. Small-cell lung carcinoma (SCLC) represents a group of highly malignant tumors giving rise to early and widespread metastasis at the time of diagnosis. However, the pancreas is a relatively infrequent site of metastasis by this neoplasm, and reports on metastatic small-cell carcinoma (SCC) in the pancreas, either of pulmonary or extrapulmonary origin, to be diagnosed by CT-scan-guided trucut biopsy (CT-TCB) are very rare. A 56-year-old man presented with a laterocervical lymphadenopathy associated to a mixed-density lung mass and a mass in the pancreatic body. CT-TCB slides from the pancreatic mass contained small, round tumor cells with extensive nuclear molding. The cytomorphological and histological diagnosis was metastatic SCC. Immunocytochemical staining showed that a variable number of neoplastic cells were positive for cytokeratin 7, TTF1, chromogranin A, and synaptophysin but negative for leukocyte common antigen and cytokeratin 20 with a very high expression of KI67. The transbronchial needle biopsy confirmed the diagnosis of an SCC. This case represents a rare metastatic lesion in the pancreas from SCLC, diagnosed by CT-TCB histological and immunohistochemical studies.

## 1. Introduction

Small-cell lung cancer (SCLC) accounts for 20–25% of all bronchogenic carcinomas and is associated with the poorest 5-year survival of all histologic types [1]. It has one of highest mortality rate among the various types of cancer and commonly metastasizes at the time of diagnosis to the lymph nodes, liver, lung, adrenal glands, brain, and bones [2, 3]. The pancreas is a relatively infrequent site of metastasis from this neoplasm [4], and reports on metastatic small-cell carcinoma (SCC) in the pancreas, either of pulmonary or extra pulmonary origin, to be diagnosed by CT-scan-guided trucut biopsy (CT-TCB) are very rare [5, 6]. However, few reports described their endoscopicultrasound (EUS) and EUS-guided fine needle aspiration (EUS-FNA) findings. Here, we report a case that was diagnosed as metastatic SCLC by CT-scan- (CT-) guided TCB histological and immunocytochemical studies of a focal pancreatic lesion.

## 2. Case Presentation

**Figure 1:**
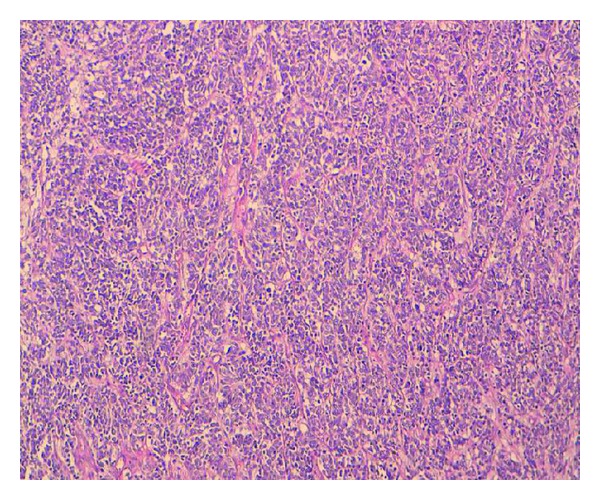
Laterocervical lymphadenopathy biopsy slide: histological features at lower magnification view (x10): proliferation of mall round tumor cells arranged in diffuse nappe with presence of some rosettes. Vascularization is spindly and endocrine type.

**Figure 2:**
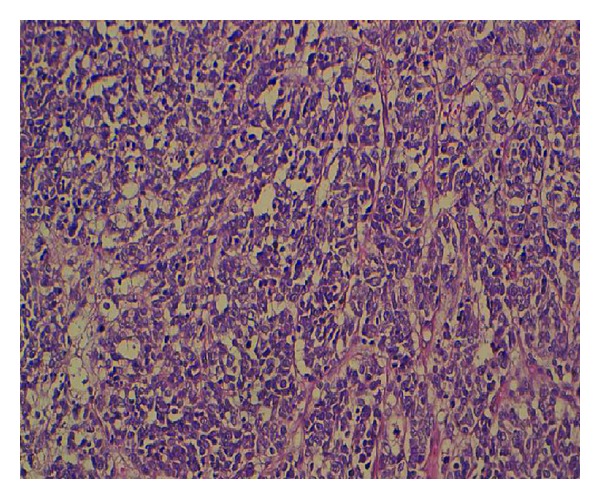
Laterocervical lymphadenopathy biopsy slide: cytomorphological features at higher magnification view: proliferation of atypical tumor cells with a high nucleocytoplasmic ratio. The nuclei of the tumor cells has salt-and-pepper-type chromatin pattern. Divers mitotic figures are noted.

**Figure 3:**
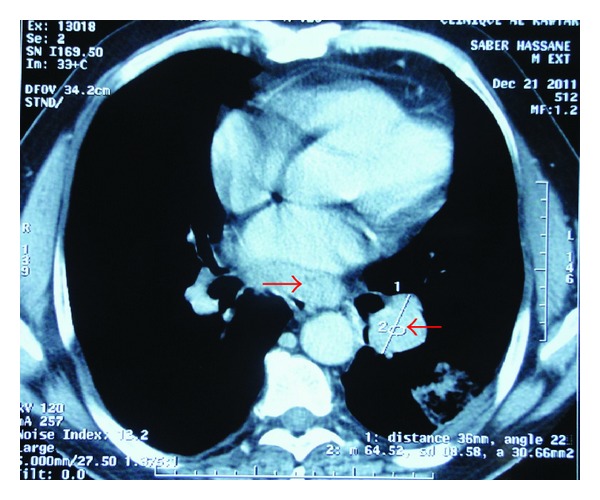
CT scan of the chest cut: extensive mediastinal lymphadenopathy with a mixed-density lung mass with irregular margins at the left hilum.

**Figure 4:**
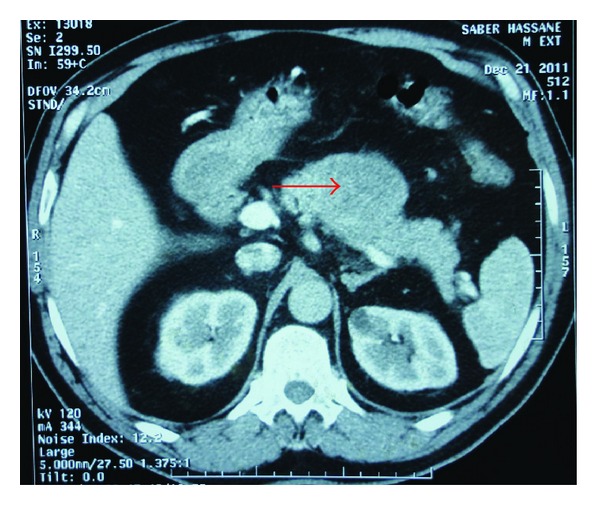
CT scan of the abdomen cut: showed a 50 mm mass in the pancreatic body, suggestive of metastasis from a primary in the lung.

A 56-year-old man, a heavy smoker, presented with a laterocervical lymphadenopathy and persistent cough. At the head and neck unit, our patient underwent a biopsy that showed a cervical lymph node metastasis of a neuroendocrine small-cell carcinoma from pulmonary origin (Figures 1 and 2) (The immunocytochemical study had demonstrated that tumor cells were positive to synaptophysin, chromogranin, CK7, and TTF1. The KI67 was expressed on 90% with negativity to CK20). Based on clinical and pathological findings, computerized tomography (CT) scan of the chest revealed extensive mediastinal lymphadenopathy with a 44 × 50 mm mixed density lung mass with irregular margins at the left hilum (Figure 3). CT scans of the abdomen and pelvic showed a left adrenal gland metastasis and a 50 mm mass in the pancreatic body, suggestive of metastasis from a primary in the lung (Figure 4).

**Figure 5:**
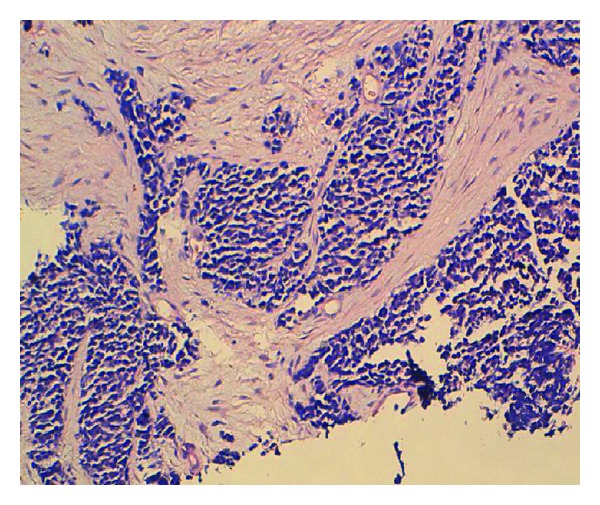
CT-scan-guided TCB slide of a pancreatic mass in a 56 year-old man: cytomorphological features at higher magnification view: small, round tumor cells with scanty cytoplasm, which showed extensive nuclear molding. The nuclei of the tumor cells were round to oval and had salt and pepper type chromatin pattern.

**Figure 6:**
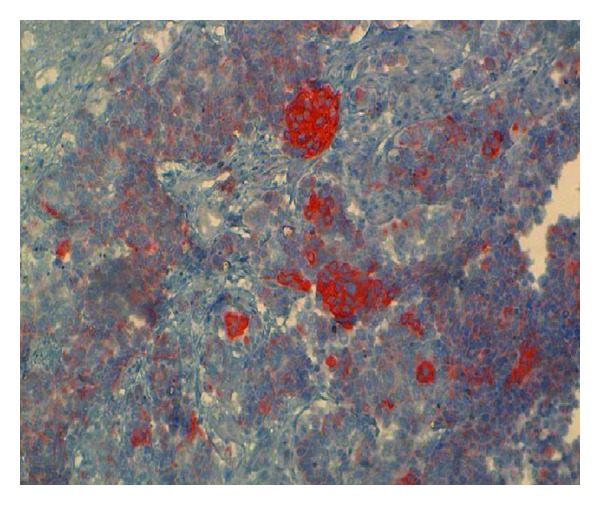
CT-scan-guided TCB slide of a pancreatic mass in a 56 year-old man. Immunohistochemical features at higher magnification view: positive reaction for cytokeratin 7 in tumor cells.

**Figure 7:**
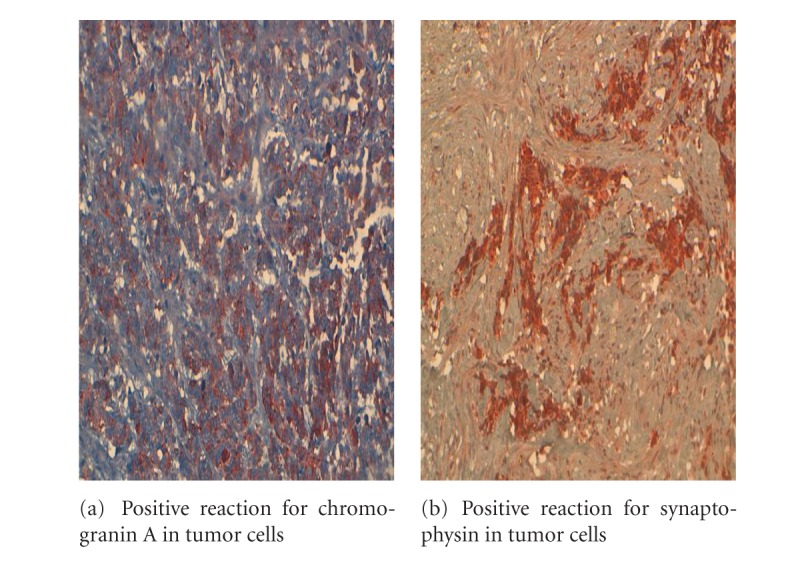
CT-scan-guided TCB slide of a pancreatic mass in a 56-year-old man. Immunohistochemical features at higher magnification view: positive reaction for chromogranin A and synaptophysin in tumor cells.

**Figure 8:**
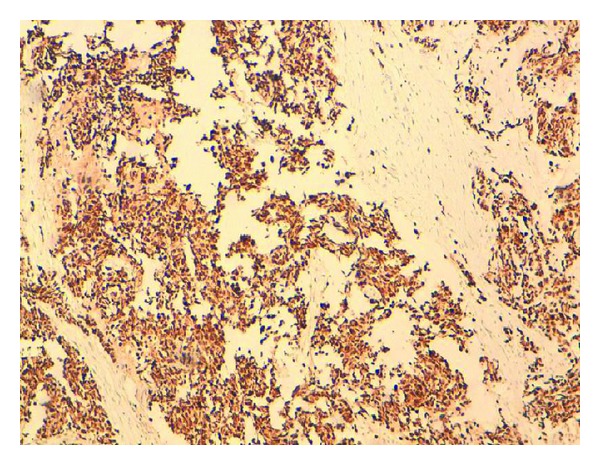
CT-scan-guided TCB slide of a pancreatic mass in a 56-year-old man. Immunohistochemical features at higher magnification view: a variable number of tumor cells expressed KI67 on 95%.

The pancreatic mass was subjected to CT-scan-guided TCB by a multidisciplinary reunion. The slides were highly cellular and contained small, round tumor cells with scanty cytoplasm, which showed extensive nuclear molding. The nuclei of the tumor cells were round to oval and had salt-and-pepper-type chromatin (Figure 5). There was an occasional microacinar formation. Rare cells with paranuclear blue inclusions and scattered apoptotic bodies were present. Most of the tumor cells were found to have a positive reaction for pan-cytokeratin, CK7 (Figure 6), TTF1, chromogranin A (Figure 7(a)) and synaptophysin (Figure 7(b)). A variable number of tumor cells expressed KI67 on 95% (Figure 8), but all were negative for leukocyte common antigen and CK20. Based on cytomorphology and immunocytochemistry, the diagnosis was metastatic neuroendocrine small-cells lung carcinoma.

The patient was subjected to transbronchial needle aspiration (TBNA) and biopsy that showed tissue fragments containing bronchial glands, closely associated with aggregates of small, round blue tumor cells. The tumor cells were positive for chromogranin A, synaptophysin, CD57, cytokeratin (Ck), and TTF1, but staining for CD 20 was negative.

 Given the conservation of performance status and renal function, the patient received a doublet regimen of metastatic first-line chemotherapy based on CDDP (100 mg/m^2^  D1 − D1 = D21) + VP16 (120 mg/m^2^D1 + D2 + D3 − D1 = D21) with a partial radiological response of all the therapeutic targets after 3 cycles of treatment. 

## 3. Discussion

Malignant lesions in the pancreas are not necessarily primary pancreatic adenocarcinomas. They can include lesions such as pseudomucinous cystadenocarcinoma, lymphoma, or pancreatic metastasis from an extrapancreatic primary tumor, which is an infrequent clinical condition and it accounts for 1, 6–11% of all pancreatic cancer cases [7, 8]. On searching medical records of 850 patients with lung cancer, Maeno  et al. [9] identified 26 (3.1%) patients with pancreatic metastasis. Yoon  et al. [10] reported 53 pathologically proven metastatic tumors of the pancreas (MTPs); the primary malignancies were renal cell carcinoma (*n* = 14), gastric cancer (*n* = 11), colorectal cancer (*n* = 5), lymphoma (*n* = 4), non-SCLC (*n* = 3), gastrointestinal stromal tumor (*n* = 2), melanoma (*n* = 2), SCLC (*n* = 2), gallbladder cancer (*n* = 2), and one case each of hepatocellular carcinoma, thymic carcinoid, liposarcoma, cholangiocarcinoma, osteosarcoma, breast cancer, duodenal cancer, and ovarian cancer. Layfield  et al. [6]  reported 17 metastatic malignancies to pancreas, which accounted for 0.73% of all pancreatic FNAs. The primary sites included eight renal cell carcinomas, four lymphomas, two squamous cell carcinomas (one from the lung and the other from the esophagus), one medullary thyroid carcinoma, one alveolar rhabdomyosarcoma, and an SCLC. The most common histological type of metastatic lung carcinoma to the pancreas is small-cell carcinoma, followed by large cell carcinoma, squamous cell carcinoma, and anaplastic bronchial carcinoma [11].

 Imaging alone, however, is not able to reliably differentiate benign or primary pancreatic tumors from metastatic lesions. For most systemic malignancies, definitive nonoperative diagnosis of pancreatic metastases may avoid unnecessary surgery for either diagnostic purposes or incorrectly suspected primary pancreatic cancer [12]. Confirming the metastatic nature of a pancreatic tumor is not an easy task, even for the pathologist. In two publications concerning ultrasound-guided pancreatic biopsies performed in patients undergoing exploration for a pancreatic mass, the diagnosis of pancreatic metastasis (PM) was retained in 3% and 6% of the patients, respectively [13, 14]. Di Stasi et al. [13] reported on 510 patients and demonstrated that the diagnosis of PM (*N* = 17) was established in 100% of the cases from the EUS-guided fine needle specimen. In their report on EUS-guided fine needle aspiration of 114 pancreatic masses, Fritscher-Ravens et al. [15] found PM in 10%. But no publication was performed in the diagnostic role of a CT-scan-guided TCB for pancreatic metastasis from a small-cell lung carcinoma.

 The SCLC includes three morphologic categories in paraffin sections: (1) SCC, (2) mixed small-cell/large-cell, and (3) combined SCC [1]. In our case, the slides were made mostly of small-cells. The neoplastic cells had scant cytoplasm and stippled or salt-and-pepper-type chromatin. Nuclear molding was conspicuous and scattered cells with paranuclear blue inclusions or apoptotic bodies were present throughout the slide. Extreme nuclear molding by clusters of small tumor cells is considered to be the most characteristic presentation of SCC; moreover, paranuclear blue inclusions, which are 1–4 mm spherical inclusions demonstrated by Romanowsky stain, may act as a diagnostically useful finding [16]. According to Arora  et al.,  [17]  nuclear molding, cell size and scant, and basophilic cytoplasm were highly sensitive and specific for distinguishing small-cell carcinoma (SCC) from Non-SCC (NSCC). Other features, such as salt-and-pepper chromatin, crush artifact, and apoptotic bodies, had also significantly high specificity; however, their low sensitivity precluded their usefulness in separating SCC from NSCC.

Difficulties may be observed in differentiating metastatic SCC from other small round cell tumors and primary pancreatic SCC from metastatic SCC. Under such situations, a panel of immunohistochemical stains can be useful in differentiating SCC from various other small-cell neoplasms [5].In practical work-up of SCC, immunohistochemistry is useful to discern SCC from high-grade lymphoma, SCC labels with antibodies to high molecular weight cytokeratins, but not with antibodies to leukocyte common antigen (CD45) [18]. In our case, the neoplastic cells in TCB slides were Ck+ and stain for LCA showed negative reaction, thus excluding the possibility of non-Hodgkin lymphoma. The most common immuno profile of metastatic SSC from a lung primary is positive staining with cytokeratin 7 and thyroid transcription factor (TTF)-1 and no reaction to Ck20 [19].However, immunohistochemical studies are found to have limited value in distinguishing a primary site from metastatic SCC [5]. According to Lin et al., [20] immunohistochemistry has not proven useful in distinguishing primary periampullary SCC from metastatic SCLC. Although a high percentage of lung primary cancer expresses TTF-1, this marker has variable positivity in patients with extra pulmonary SCC, [20] and the expression of TTF-1 has not been well explored in pancreatic SCC, with only one negative reported case in the literature to date [21].Moreover, cytokeratin (CK7 and CK20) switching can occur in the natural history of pulmonary SSC [19]. 

Pulmonary neuroendocrine tumors (NETs) are traditionally described as comprising a spectrum of neoplasms, ranging from low-grade typical carcinoids (TC) via the intermediate-grade atypical carcinoids (AC) to the highly malignant small-cell lung cancers (SCLCs) and large cellneuroendocrine carcinomas. Recent data, however, suggests that two categories can be distinguished on basis of molecular and clinical data, the high-grade neuroendocrine (NE) carcinomas, and the carcinoid tumors [22]. Histopathologically, high-grade NE lung tumors are characterized by high mitotic and proliferative indices, while carcinoids are defined by maximally 10 mitoses per 2 mm (2) (10 high-power fields) and rarely haveKi67-proliferative indices over 10% [22]. On the other hand, mutations of the multiple endocrine neoplasia type 1 (MEN1) gene are restricted to carcinoid tumors. However, in our case, the KI67 proliferative index was very high. 

## 4. Conclusion

Pancreatic metastases are an important cause of focal pancreatic lesions and may occasionally be discovered during CT staging. Use of immunocytochemistry, when available, may help to confirm a suspected diagnosis.

In our case, the clinical suspicion was SCLC. Histology and immunohistochemical studies also favored the primary tumor as SCLC. The pancreatic CT-scan-guided TCB was diagnosed as metastatic SCC, which was positive for pan-Ck, chromogranin A, and synaptophysin. However, the immunohistochemical panels included Ck7, Ck20, TTF-1, and KI67, which had further confirmed the primary lung cancer.
